# Root‐Filled Teeth With and Without Pain in a Cohort of Individuals Scheduled for Regular Dental Check‐Ups. A Matched Case–Control Study

**DOI:** 10.1111/joor.70089

**Published:** 2025-10-28

**Authors:** Jakob Jonsson Sjögren, Maria Pigg, Alf Eliasson, EndoReCo, Lars Bjørndal, Victoria S. Dawson, Helena Fransson, Fredrik Frisk, Peter Jonasson, Merete Markvart, Dan Sebring, Emma Wigsten, Thomas Kvist

**Affiliations:** ^1^ Faculty of Medicine and Health Örebro University Örebro Sweden; ^2^ Department of Endodontics, Faculty of Odontology Malmö University Malmö Sweden; ^3^ Department of Endodontology, Institute of Odontology at the Sahlgrenska Academy University of Gothenburg Gothenburg Sweden

**Keywords:** diagnosis, endodontics, facial pain, pain, root canal therapy

## Abstract

**Background:**

An overwhelming majority of root‐filled teeth are asymptomatic, despite commonly exhibiting radiological signs of apical periodontitis (AP). When symptoms prevail, several sources are conceivable. This case–control study investigates underlying causes of pain from root‐filled teeth.

**Objectives:**

The aims were to (i) compare painful root‐filled teeth with root‐filled teeth without pain matched on sex, age, jaw and tooth type, and (ii) explore patient‐ and tooth‐related factors that may explain the pain.

**Methods:**

Adult participants (≥ 20 years) with root‐filled teeth were investigated through anamnestic, clinical and radiographic examinations. Analyses compared painful to pain‐free teeth statistically, and possible origins of pain were indicated. Clinical data and periapical radiographs were used to identify the presence of AP.

**Results:**

*Tenderness to percussion* and *apical palpation*, *probing depth* ≥ *6 mm*, *swelling* and *apical radiolucency* (*p* = 0.002–0.040) were more common with painful teeth, while *density* and *length of root filling*, *unfilled canals*, *fracture of root* and *positive screening for TMD* (3Q/TMD) did not differ between groups (*p* = 0.074–0.63). Among the 55 symptomatic teeth, AP was identified in 48 with varying diagnostic certainty. Indication of temporomandibular disorder (TMD) was present in 15 teeth, and marginal periodontitis (MP) in eight teeth. For six teeth, none of the three conditions could be identified.

**Conclusions:**

*Tenderness to percussion* and *apical palpation*, *swelling*, *pocket depth* ≥ *6 mm*, and *apical radiolucency* were more common with painful teeth. The pain was most frequently associated with AP, but pain due to TMD and MP may occur. For 10% of the root‐filled teeth, no findings could explain the symptoms.

## Graphical abstract

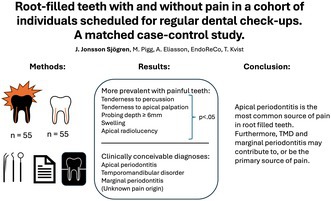

## 1 Introduction

In European countries, approximately 60% of the adult population has at least one root‐filled tooth [1]. Among these, 25%–50% exhibit radiolucencies indicating apical periodontitis (AP) [2, 3, 4, 5, 6]. Approximately 5% are symptomatic ≥ 6 months after the root canal treatment (RCT) [7]. It is reasonable that teeth with AP may be symptomatic. However, several studies have reported that root‐filled teeth without signs of periapical pathology may occasionally also be painful [8, 9, 10]. Consequently, the presence of AP appears to be neither necessary nor a sufficient explanation for symptoms from root‐filled teeth.

Pre‐operative pain interfering with daily activities, pain made worse with stress, and a diagnosis of symptomatic AP are associated with an increased risk of severe post‐operative pain up to 1 week after RCT [11]. Also, chronic pain problems and previous painful treatment(s) in the orofacial area are risk factors for persistent pain [12]. With time, such pain often gradually decreases or disappears without any intervention [13, 14]. However, some root‐filled teeth remain painful over time [15].

In a previous paper, we reported on 53 patients with 62 root‐filled teeth, identified in regular dental check‐up visits [8]. The average pain duration was > 2 years, and the average pain intensity was reported to be low, on average 2.1 on a 0–10 Numeric Rating Scale (NRS) and had a low negative impact on daily life. Although the associated clinical and radiographic findings indicated AP as the most frequent cause of pain, the origin of pain from these root‐filled teeth remained partly unexplained.

The aim of this study was to compare, in detail, painful root‐filled teeth with root‐filled teeth without pain, matched on sex, age, jaw and tooth type. An additional aim was to explore patient‐ and tooth‐related factors that may explain the pain.

## 2 Materials and Methods

This study follows the guidelines of PROBE 2023 (Preferred Reporting Items for OBservational studies in Endodontics) [16]. The PROBE checklist, Supporting Information S1, is based on the STROBE statement (STrengthening the Reporting of OBservational studies in Epidemiology) [17].

### 2.1 Study Design and Population

This case–control study used cross‐sectional data collected in 2015 at 23 Public Dental Service clinics in Region Örebro County, Sweden. The patients who were scheduled for a regular check‐up in April 2015 were screened beforehand for the presence of root‐filled teeth by examination of all earlier digital radiographs. Patients having ≥ 1 root‐filled tooth were informed of the study before the appointment in writing and again orally at the appointment, and thereafter invited to participate. Before data collection took place, one of the authors (J.J.S.) informed the clinic personnel about the details of the procedure to ensure methodological consistency. The data were collected concurrently with the regular check‐up, and the same dentist conducted both the check‐up and data recording. The pool of patients in the cross‐sectional study from which the participants were drawn consisted of 550 patients with 1256 root‐filled teeth [8]. The consent forms are stored in a locked room at the Public Dental Service.

### 2.2 Ethical Approval

The regional Ethics Review Board in Uppsala, Sweden (daybook no. 2014/197) approved the study.

### 2.3 Participants and Data Collection

**FIGURE 1:**
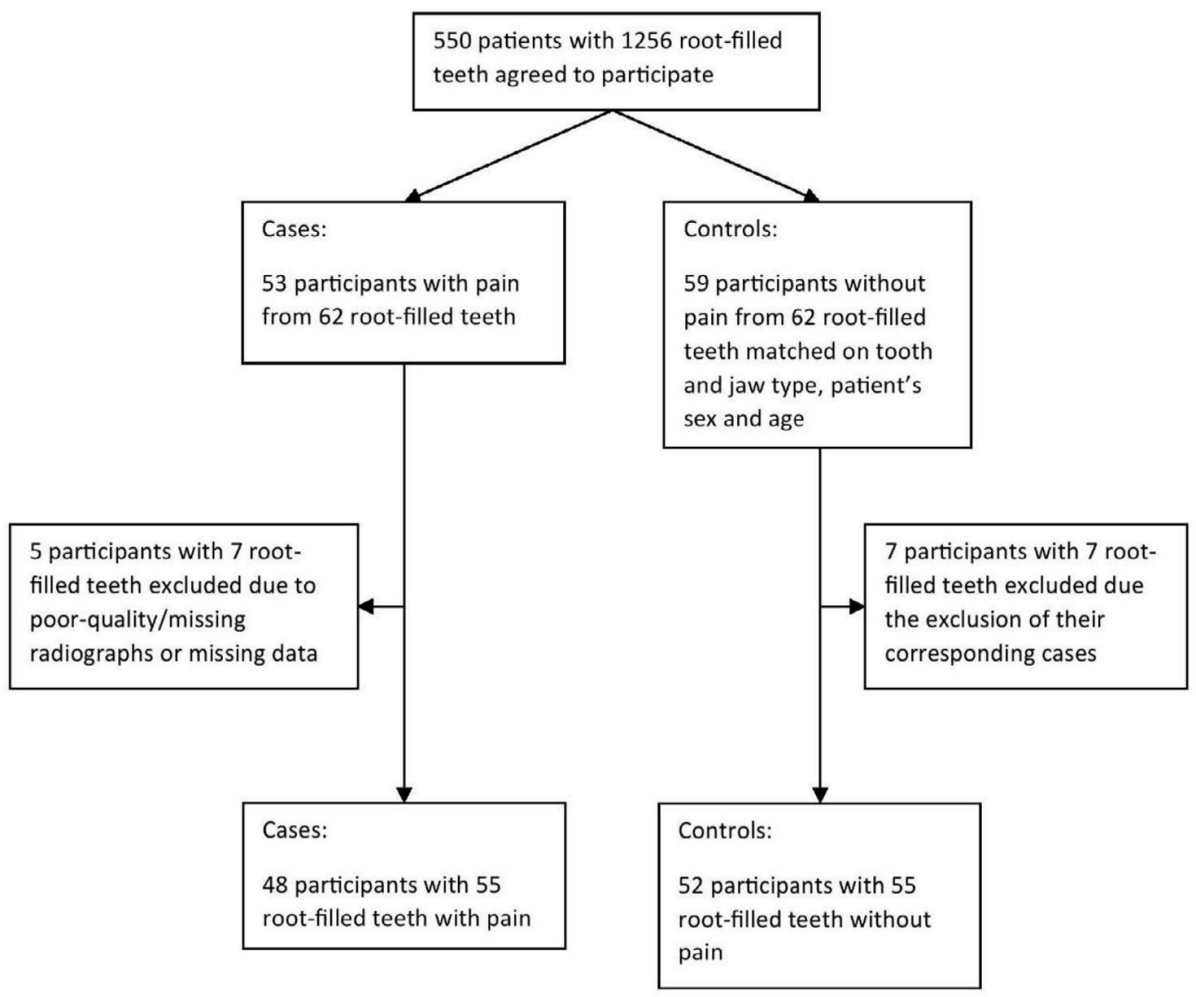
Flow‐chart of inclusion and exclusion of participants. Cases and controls were included from a data collection on root‐filled teeth that took place in 2015.

All patients with a root‐filled tooth causing pain or discomfort were included. A case was defined as a patient with a painful or uncomfortable root‐filled tooth. From the same pool, one control patient with an asymptomatic tooth was included for every case. The controls were matched on tooth type (incisor/canine, premolar or molar), patient's sex, patient's age (within 10 years) and jaw type (maxilla or mandible). The inclusion and exclusion of participants are displayed in Figure 1.

The data collected were:

- Self‐reported anamnestic data: All participants filled out a questionnaire with questions on patient‐level (for cases and controls):
  ○ Presence of symptoms from any root‐filled tooth, according to the response to the initial question ‘Have you experienced pain or discomfort (swelling, tenderness on chewing or any other problem) from any of your root‐filled teeth at any time during the last 3 months?’ (YES/NO) This response determined the patient as a case or a control.
  ○ General health; the participants reported whether they suffered from either diabetes, cardiovascular disease, gastrointestinal disorder, rheumatic disease, neurological disease or psychiatric disorder. In the analysis, the answer was dichotomised to either ‘healthy’ or ‘≥ 1 health concern’.
  ○ Symptoms of temporomandibular disorder (TMD), according to the participant's response to the two 3Q/TMD screening questions [18] related to pain (the third question concerns locking of the jaw and was deemed not relevant here).
    Q1: *Do you have pain in your temple, face, jaw or jaw joint once a week or more?* (YES/NO).
    Q2: *Do you have pain once a week or more when you open your mouth or chew?* (YES/NO).
  ○ Presence of chronic bodily pain, lasting at least 3 months, according to the response to the question ‘For at least the last 3 months, have you experienced pain in more than one area of your body during at least 4 days each week?’ [19] (YES/NO).
- Clinical data: *Tenderness to percussion* and *apical palpation* (YES/NO), greatest periodontal *probing depth* (≤ 5 mm/≥ 6 mm) [20], *sinus tract* (YES/NO), *swelling* (YES/NO) and *quality of the coronal restoration* (GOOD/POOR; based on presence of caries, insufficient marginal integrity, temporary/leaking restoration or root filling exposed).
- Radiographic data: One or two intraoral periapical radiographs of the root‐filled tooth were exposed. One radiograph was sufficient if an apical radiolucency was observed, and a second radiograph was exposed if the apical contour(s) were deemed normal by the dentist collecting data. This was done in order to increase the sensitivity [21]. Three specialists in Oral and Maxillofacial Radiology reviewed the radiographs independently under optimal conditions with no information on the presence of symptoms or clinical presentation of the teeth. The majority principle ruled the outcome; if two of the three specialists made the same assessment, the variable was recorded as such. Table 1 shows the variables assessed. Observer agreement was calculated.

**TABLE 1:** The registrations and codes used by the three observers when assessing the radiographs.

Variable assessed	Registrations and codes	Comments
Apical status	1 = Normal, apical periodontal ligament space not more than double width as compared to other parts of the root 2 = Widened periodontal ligament space, ≤ 1 mm 3 = Apical radiolucency, > 1 mm width	If lamina dura was not visible, the tooth was classified as healthy Codes 1 and 2 were merged into the category healthy Multirooted teeth were classified as the root with the worst condition
Density of root filling [22]	0 = Complete obturation, no lateral or apical lumen visible 1 = Incomplete obturation, visible canal lumen lateral or apical of the root filling	
Distance between the end of root filling to radiographic apex [23]	0 = 0–2 mm short of radiographic apex 1 = > 2 mm short of radiographic apex 2 = Excess	The categories deficit and excess were merged into one group in the analyses
Missed canals	0 = No 1 = Yes	
Fracture of the root	0 = No 1 = Yes	
Presence of post	0 = No 1 = Yes	
Length of root filling under the post	Measured in mm	
Coronal restauration^a^	0 = Adequate, radiographically sealed on both sides 1 = Inadequate, deficiencies in the form of marginal integrity, surplus marginal integrity or no coronal integrity	If one side is inadequate, the entire restoration was considered inadequate
Type of restoration	0 = Single crown 1 = Crown in bridge 2 = Direct restoration	
Secondary carious lesion^a^	0 = No 1 = Yes, caries is visible in relation to the restoration	
Marginal breakdown of bone	0 = No 1 = ≤ 1/3 of root length 2 = 1/3 to 2/3 of root length 3 = ≥ 2/3 of root length	The worst site counts Categories 1, 2 and 3 were merged into one group in the analyses and contrasted with group 0
Number of neighbouring teeth	0 = No 1 = One neighbouring tooth 2 = Two neighbouring teeth	

^a^ Adaptation by Kirkevang et al. [24].

Clinical and radiographic data were reported at the tooth level.

### 2.4 Statistical Analyses

The data were entered in Excel (Microsoft Corporation, Redmond, WA, USA) and imported into the statistical software. For descriptive statistics (number, percentage, mean and standard deviation [SD]) concerning demographic and tooth‐related data, IBM SPSS version 29 (IBM, Armonk, NY, USA) was used. Fisher's exact test (lowest one‐sided *p*‐value multiplied by 2), linear mixed‐effects model, and generalised mixed effects model were performed with SAS/STAT Software, version 9.4 of the SAS System for Windows (SAS Institute, Cary, NC).

No power calculation was performed.

The linear or generalised linear mixed effects models were used with subjects as a random effect, accounting for intraindividual correlations in repeated measures data (multiple teeth per subject). Linear mixed effects models were used for continuous variables. Non‐normal (positively skewed) variables were log‐transformed prior to analysis, and results were presented as fold changes between groups, calculated by exponentiating the regression coefficients on the log scale. Robust standard errors (HC3 method) were employed to account for possible violations of distributional assumptions. Binary variables were analysed using a logit link (i.e., using mixed effects logistic regression) and results were presented using odds ratios (OR). The OR signifies the odds of experiencing pain from a tooth with the independent variable being positive. In cases of non‐convergence (e.g., due to too few events), OR were estimated using the Firth correction. All analyses were adjusted for matching variables: age group (categorical), sex, tooth type, and jaw type. All estimates are presented with 95% confidence intervals (CI). For interobserver agreement on the variable apical status, kappa statistics were used. *κ* ≤ 0.20 equals slight agreement, 0.21–0.40 equals fair, 0.41–0.60 moderate, 0.61–0.80 substantial and 0.81–1.0 equals almost perfect agreement [25]. Some variables have missing data; hence, the valid numbers differ between variables. No imputation was performed for missing data. All significance tests were two‐sided and conducted at the 0.05 level of significance.

### 2.5 A Clinical Reasoning Approach to Data

Based on the anamnestic, clinical, and radiographic findings, the painful teeth were divided into four main categories of plausible origin of pain: I. AP, II. MP (marginal periodontitis), III. TMD and 0. Unknown. A tooth could qualify for inclusion in one or multiple categories. Colour coding was used for categorisation (Supporting Information S2). The findings were categorised as: radiographic and/or clinical signs (*apical radiolucency*, *sinus tract*, *apical swelling*, *apical tenderness* or *tenderness to percussion*) directly and (*poor root filling*, *suboptimal length of root filling*, *untreated canals*, *poor restoration* or *caries*) indirectly indicating AP, signs of MP (*greatest probing depth* ≥ *6 mm*, *marginal breakdown of bone*), and signs of TMD (positive *screening for painful TMD*).

I. Apical periodontitis (AP)

The category AP was subcategorised into four different classes in an order of certainty of diagnosis, in a lexicographical order (1, 2, 3 and 4) where a higher classification trumps a lower one. The categories were defined as follows:

AP‐1. Definite AP

*Apical radiolucency* + ≥ 1 positive finding of *sinus tract*, *apical swelling*, *apical tenderness* or *tenderness to percussion*.

AP‐2. Probable AP

*Apical radiolucency* without clinical findings or no apical radiolucency but ≥ 2 positive findings of *sinus tract*, *apical swelling*, *apical tenderness* or *tenderness to percussion*.

AP‐3. Possible AP

No apical radiolucency but ≥ 1 of the positive findings of *sinus tract*, *apical swelling*, *apical tenderness* or *tenderness to percussion*.

AP‐4. Conceivable AP

Not qualifying for either of 1, 2 or 3, but with ≥ 1 indirect radiological sign possibly suggesting AP: *poor root filling*, *suboptimal length of root filling*, *untreated canals*, *poor restoration* or *caries*.

II. Marginal periodontitis (MP)

MP: Teeth with both the *greatest probing depth* ≥ *6 mm* and a *marginal breakdown of bone* ≥ one‐third of the root length observed on the radiograph.

III. Temporomandibular disorder (TMD)

TMD: A positive answer to Q1 or Q2 of the *screening questions for TMD*.

0. Unknown

Other diagnoses may be the origin of pain. No finding qualifies for any of the categories I, II or III.

## 3 Results

This study included 100 participants in total, 48 contributing with 55 painful teeth and 52 contributing with 55 asymptomatic teeth. The mean age was 56.8 years (SD 13.8 years). Women comprised 62% of the participants. Maxillary teeth constituted 56.4% of the sample. Incisors/canines constituted 21.8% of the teeth, 16.4% were premolars and 61.8% were molars.

### 3.1 Statistical Analyses

#### 3.1.1 Patient Characteristics

About half of the cases and controls reported having ≥ 1 general health concern and about one‐third reported chronic bodily pain. A third of the cases compared to a tenth of the controls reported a positive answer to any of the screening questions. The distribution of the patient characteristics and a comparison between cases and controls are seen in Table 2.

**TABLE 2:** Comparison of patient characteristics between cases and controls, patient level.

	Cases	Controls	*p*	Missing *n*
	*n* = 48	*n* = 52		Cases/controls
General health				
≥ 1 general health concern; *n* (%)	21 (53.8)	18 (48.6)	0.820	9/15
Screening questions for TMD (3Q/TMD)^a^				
Q1 or Q2: *Do you have pain in your temple, face, jaw or jaw joint once a week or more?* OR *Do you have pain once a week or more when you open your mouth or chew?* Yes; *n* (%)	13 (27.7)	6 (11.5)	0.074	1/0
Q1: *Do you have pain in your temple, face, jaw or jaw joint once a week or more?* Yes; *n* (%)	7 (14.9)	4 (7.7)	0.410	1/0
Q2: *Do you have pain once a week or more when you open your mouth or chew?* Yes; *n* (%)	9 (18.8)	3 (5.8)	0.089	0/0
For at least the last 3 months, have you experienced pain in more than one area of your body during at least 4 days each week? Yes; *n* (%)^b^	19 (39.6)	15 (28.8)	0.360	0/0

*Note:* *p* < 0.05 was considered statistically significant.

Abbreviation: TMD, temporomandibular disorder.

^a^ Lövgren et al. [18].

^b^ Nixdorf et al. [19].

#### 3.1.2 Clinical Findings

About 4 in 10 of the symptomatic teeth exhibited *tenderness to percussion*, and 1 in 10 was reported to be *tender on apical palpation*. Almost 1 in 5 had a *greatest probing depth* ≥ *6 mm*; *swelling* was seen in 1 in 10 teeth. These were all statistically more common with the painful teeth. The details can be seen in Table 3.

**TABLE 3:** Comparison of clinical and radiographic characteristics among painful and asymptomatic teeth. OR stands for the odds of experiencing pain from a tooth with the independent variable being present.

	Cases	Controls	*p*	OR (95% CI)	Missing *n*
Clinical characteristics					
Tenderness to percussion, *n* (%)^a^	21 (38.2)	0 (0)	**0.002**	**67.72 (4.79–956.86)**	0
Tenderness to apical palpation, *n* (%)^a^	7 (12.7)	0 (0)	**0.030**	**15.91 (1.31–192.52**)	0
Greatest probing depth ≥ 6 mm, *n* (%)	10 (18.5)	1 (1.8)	**0.008**	**9.50 (1.80–50.04)**	1
Sinus tract, *n* (%)^a^	3 (5.5)	0 (0)	0.096	7.92 (0.69–90.74)	0
Swelling, *n* (%)^a^	7 (12.7)	0 (0)	**0.029**	**16.46 (1.33–204.07)**	0
Poor quality of coronal restoration, *n* (%)	8 (15.7)	10 (18.9)	0.650	1.35 (0.28–6.48)	6
Radiographic characteristics					
Apical status^b^			**0.040**	**3.76 (1.08–13.01)**	1
Apically healthy, *n* (%)	35 (63.6)	46 (85.2)			
Apical radiolucency, *n* (%)	20 (36.4)	8 (14.8)			
Density of root filling^b^			0.210	0.61 (0.28–1.33)	2
Complete obturation, *n* (%)	31 (57.4)	24 (44.4)			
Incomplete obturation, *n* (%)	23 (42.6)	30 (55.6)			
Length of root filling^b^			0.320	0.63 (0.23–1.75)	0
0–2 mm from apex, *n* (%)	34 (61.8)	28 (50.9)			
> 2 mm short of apex or excess, *n* (%)	21 (38.2)	27 (49.1)			
Unfilled canals, *n* (%)^b^	1 (1.8)	5 (9.1)	0.091	0.22 (0.04–1.28)	0
Fracture of root, *n* (%)^b^	2 (3.6)	0 (0)	0.170	4.99 (0.51–49.05)	0
Post and core, *n* (%)^b^	8 (14.5)	18 (32.7)	0.054	0.24 (0.06–1.03)	0
Length of root filling under post and core (log), median (IQR)^c^, ^d^	1.61 (1.4–1.9)	1.55 (1.1–1.8)	0.840	FC = 1.10 (0.01–128.22)	1
Adequate coronal seal^b^ according to radiograph			0.510	1.52 (0.37–6.34)	
Adequate seal, *n* (%)	46 (83.6)	48 (87.3)			
Inadequate seal, *n* (%)	9 (16.4)	7 (12.7)			
What restoration type^b^			0.850	1.10 (0.32–3.81)	0
Single crown/crown in bridge, *n* (%)	14 (25.5)	15 (27.3)			
Direct restoration, *n* (%)	41 (74.5)	40 (72.7)			
Secondary caries^b^			0.260	3.05 (0.36–26.21)	0
No, *n* (%)	49 (89.1)	52 (94.5)			
Yes, *n* (%)	6 (10.9)	3 (5.5)			
Marginal breakdown of bone^b^			0.990	0.99 (0.35–2.85)	0
No, *n* (%)	18 (32.7)	17 (30.9)			
1/3 or more of root length, *n* (%)	37 (67.3)	38 (69.1)			
Number of neighbouring teeth^b^			0.990	1.00 (0.37–2.71)	1
0 or 1, *n* (%)	19 (34.5)	18 (33.3)			
Two neighbouring teeth, *n* (%)	36 (65.5)	36 (66.7)			

*Note:* *p* < 0.05 was considered statistically significant; significant results are in bold type.

Abbreviations: CI, confidence interval; FC, fold change; IQR, interquartile range; OR, odds ratio; SD, standard deviation.

^a^ Firth correction due to few events.

^b^ Mixed effects logistic regression.

^c^ Linear mixed effects model.

^d^ Log transformed. Median (IQR). Fold change presented.

#### 3.1.3 Radiographic Findings

The range of observer agreement between the three radiologists for the variable apical status was *κ* 0.525–0.755, which corresponds to moderate to substantial agreement [25].

The only significant difference between the symptomatic and asymptomatic teeth among the radiographic findings was apical status. A third of the symptomatic teeth and a sixth of the asymptomatic teeth had *apical radiolucency*. The details of the radiographic characteristics are seen in Table 3.

### 3.2 Clinical Reasoning

**FIGURE 2:**
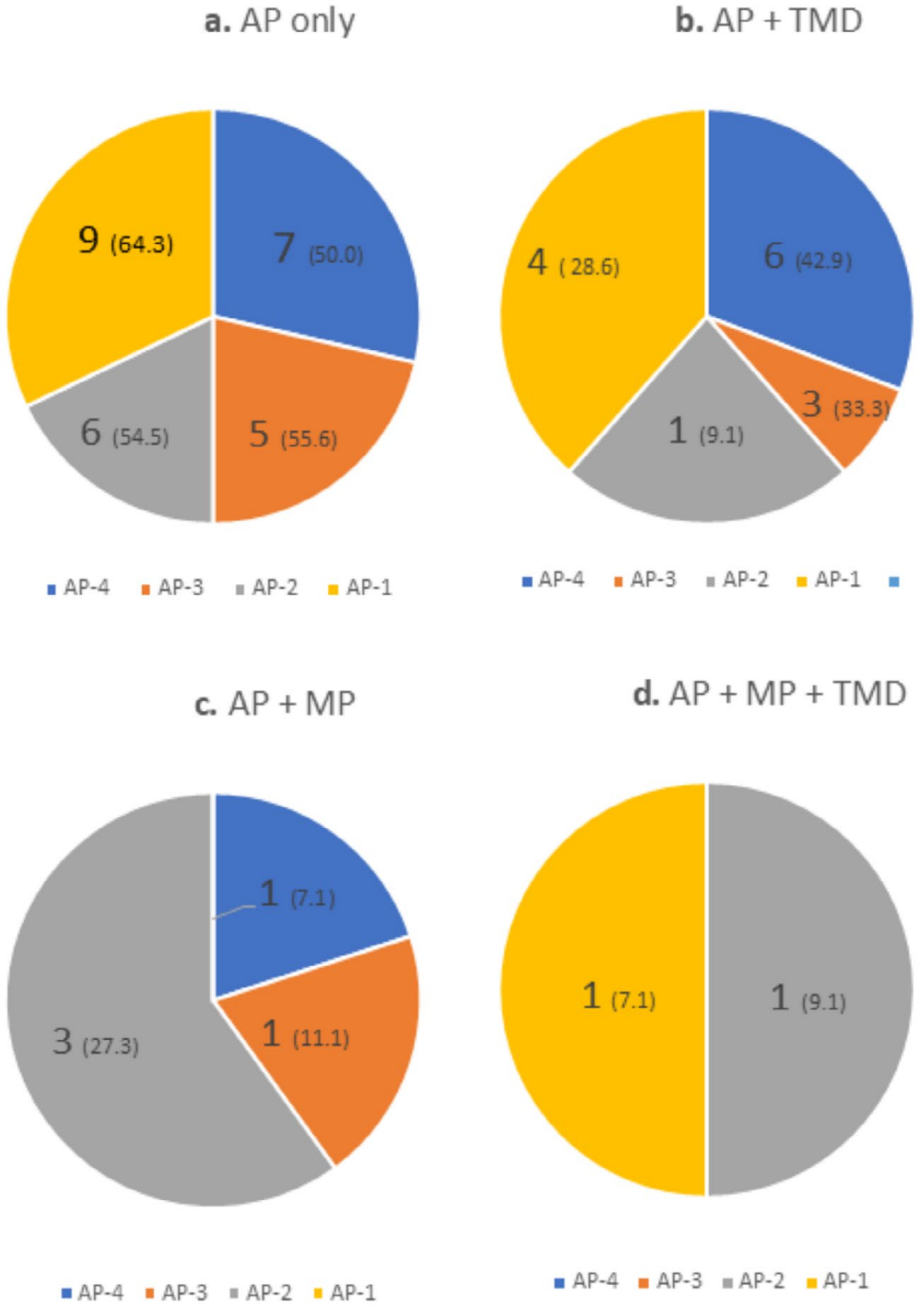
(a–d) Distribution of specific combinations of possible conditions; *n* (%). Colours denote the level of certainty of the AP diagnosis, and percentages denote proportions within each level. (a) AP only, total *n* = 27 (56.3%); (b) AP + TMD, total *n* = 14 (25.5%); (c) AP + MP, total *n* = 5 (10.4%); (d) AP + MP + TMD, total *n* = 2 (4.2%). Excluded from the list are one tooth with MP as the only condition and six teeth not qualifying for any condition. AP, apical periodontitis; AP‐1, definite AP; AP‐2, probable AP; AP‐3, possible AP; AP‐4, conceivable AP; MP, marginal periodontitis; TMD, temporomandibular disorder.

Supporting Information S2 displays the findings of all matched pairs in detail. Of the 55 symptomatic teeth, *n* = 14 (25.5%) presented with definite AP, *n* = 11 (20.0%) with probable AP, *n* = 9 (16.4%) with possible AP and *n* = 14 (25.5%) with conceivable AP. Twenty‐seven teeth (56.3%) exhibited only the indication of AP with various certainty, while the remaining 21 teeth (43.7%) had ≥ 1 other finding as a possible origin of pain. Figure 2 lists the specific combinations of conditions. Fourteen painful teeth (29.2%) were found in individuals with indications of TMD. Among these, AP was definitely or probably present in the painful tooth in five teeth (33.3%). In the remaining cases, for three teeth (20.0%), AP was possibly present, but for the remaining six teeth (40.0%), the level of diagnostic certainty was only conceivable AP. Eight painful teeth (14.5%) presented both radiological and clinical signs of MP. In one tooth, MP was the only recording of disease, while the remaining seven also presented signs of AP with varying certainty, one (12.5%) definite, four (50.0%) probable, one (12.5%) possible, and one (12.5%) conceivable. Of these eight teeth with signs of MP, two also had signs of both AP and TMD. Six (10.9%) of the painful root‐filled teeth did not qualify in any category.

## 4 Discussion

The findings of this study corroborate that AP is the plausible origin of pain in the majority of root‐filled teeth presenting with pain in a cohort of adult individuals attending regular check‐ups. Clinical and radiological findings indicating AP were more common in cases than controls, and for 60% of the painful teeth, AP was the plausible origin of pain. TMD and MP were found to be likely contributing factors to the pain in another 30% of painful root‐filled teeth. For approximately 10% of the painful root‐filled teeth, no clinical or radiological findings could explain the symptoms.

Studies of pain from root‐filled teeth report that roughly ¼ and ⅖, respectively, of the investigated teeth have AP [9, 26]. In this study, the proportion of teeth with definite AP is on the same level as reported by Nixdorf and colleagues [9]. In the other studies, the diagnostics were dichotomous (AP or not), and the definition of disease was strict, the same as for the group ‘definite AP’ reported here. However, close to 9 out of 10 investigated teeth in this study have AP of varying diagnostic certainty, and with looser criteria, the probability of diagnosing AP increases.

In this study, there were no statistical differences between cases and controls regarding positive responses to questions formulated to screen for TMD. However, in the clinical reasoning part of the study, we found indications that AP was often accompanied by a positive response to the screening questions. This is in agreement with a recent study on the prevalence of painful TMD among patients with painful teeth referred for endodontic treatment [27]. In their study, pulpal or periapical pain and painful TMD occurred simultaneously without affecting each other in 26% of the patients. Twenty per cent of the patients had both TMD and endodontic pain, with TMD contributing to the tooth pain, and for 8% the perceived tooth pain was exclusively related to TMD [27]. The study populations differ between the studies since the present study included patients *not* seeking treatment, cross‐sectionally identified in general practice dentistry, while Daline and coauthors included patients referred for specialist treatment [27]. In conclusion, TMD as a cause for pain from root‐filled teeth cannot be ruled out.

Pain from root‐filled teeth due to marginal periodontitis is a possibility [28] but has not been extensively reported. The definition for MP in this study must be interpreted with some caution. Bleeding on probing is a fundamental part of periodontal diagnostics [29] and the examinations in this study did not include this parameter. Hence, the report of MP is uncertain, but only one case in this study exclusively received the categorisation MP.

Based on the examinations, no pain origin could be identified for six of the painful teeth. Several potential diagnoses not explored in this study may account for the pain, and additional examinations would be needed to identify these underlying causes. These would include assessment for neuropathic pain [30, 31, 32], examination of neighbouring and ipsilateral teeth for potential pain referral [9, 33], sinusitis [34], a comprehensive examination according to DC/TMD (Diagnostic Criteria for TMD) [35, 36] and assessment for possible neurovascular (headache‐related) pain [37, 38, 39]. An additional diagnostic possibility is pain of unknown origin, persistent idiopathic dentoalveolar pain [28]. However, as the data collection was conducted in general practice during a routine check‐up with limited time availability, these examination methods were not feasible, and in some cases, exclusion is not within the competency of a general dentist. The diagnoses of AP were based on clinical and radiographic findings. *Tenderness to percussion* and *apical tenderness* have often been associated with AP [40, 41] but also with other pain diagnoses [33, 34, 35]. In particular, tenderness to percussion has been shown to be an unreliable sign of AP. Furthermore, one study found that teeth with probable non‐odontogenic pain exhibited more frequent tenderness to apical palpation than those with odontogenic pain [9]. Therefore, such findings may be better interpreted as a change in somatosensory function rather than a specific disease marker, thus offering limited diagnostic value in determining the origin of pain in root‐filled teeth.

This study included all teeth causing symptoms (pain or discomfort) from the data collection in 2015 in order to have the most robust analysis possible. However, some had to be excluded due to missing information (poor‐quality/missing radiographs or missing data).

No power calculation preceded the data collection. This entails a risk of Type II error, i.e., non‐significant results being false negatives. Examples could be *positive screening for TMD* (3Q/TMD) as TMD has been associated with painful root‐filled teeth [42] or variables associated with failed RCTs, *incomplete obturation*, or *suboptimal length of root filling* [43, 44] or *coronal seal* [45]. These variables could be investigated in future studies. However, in the clinical reasoning approach to the data, where statistics were not used, the study population was large enough to show a detailed picture of symptomatic root‐filled teeth by using all the included variables and highlighting the complexity in diagnostics.

Our analyses are strongly dependent on the radiological diagnoses. The radiographic review was thorough. Three experienced specialists in Oral & Maxillofacial Radiology reviewed the radiographs, and in disagreement, the diagnosis was decided by majority principle. The reviewers worked under optimal viewing conditions independently of each other and were blinded to the presence or absence of pain, factors that reduce the risk of bias [46].

The validity of a radiolucency as a sign of AP must be questioned. The data are cross‐sectional, and therefore, it is not possible to tell if the *apical radiolucency* represents AP as an ongoing disease or as a state of healing. Additionally, it is well known that intraoral radiographs are less sensitive than Cone Beam Computed Tomography (CBCT) in detecting apical radiolucency. However, when using histology as the reference, CBCT has shown lower specificity for diagnosing apical periodontitis (AP) in root‐filled teeth compared to non‐root‐filled teeth [47]. If additional CBCT scans had been performed in our study, they would likely have shown more apical radiolucencies, but it remains unclear how many of these would have represented ongoing AP [48].

In this study, *incomplete obturation*, *length of root filling* > *2 mm short of apex or excess*, *unfilled canals*, *inadequate seal* and *secondary caries* were used as indirect indicators of AP. In nine cases, only findings *conceivably* (only one indirect sign diagnosed) indicating AP as the origin of pain were made (Figure 2). It could be argued that these teeth should rather be classified together with the six cases where no explanatory finding could be made. In that scenario, 15 (27.3%) of the painful teeth would qualify for the category of ‘unexplained’ pain. For teeth with a suspected other origin of pain, additional imaging and clinical examinations are needed for diagnosis [10].

The possibility of referred pain from TMD as a cause of pain in this study is also surrounded by uncertainty. No complete DC/TMD examination was performed on the participants, so the actual TMD diagnoses are not known, but a positive response to question one or two of 3Q/TMD has a sensitivity and specificity > 0.8 for a DC/TMD pain diagnosis [18]. However, the 3Q/TMD has not been validated in a tooth pain population, and this entails the risk of false positive answers. Another instrument developed to screen for TMD is the TMD Pain Screener, which comprises six questions and has good validity. This instrument was recently validated for use in a population with endodontic pain [42] but was not available at the time for our data collection.

The validity of the results is an important aspect. The data were collected with standard practice examination methods, and the clinical personnel were informed beforehand about how the study was to be performed. Therefore, the data were collected under similar conditions for all patients, and the internal validity of the data must be considered good.

External validity is ensured by the setting; this is a practice‐based study from general practice, where most root canal treatments are performed, and the participants are regular attendees of dental care, like the majority of the population, which is one of the strengths of the study.

### 4.1 Future Research

We suggest further studies exploring the origin of persistent pain in root‐filled teeth that involve more extensive anamnestic, clinical, and radiological examinations, since standard methods appear to be inadequate to identify the pain origin in a proportion of cases. Also, in order to strengthen the causative relationship between subjective pain in root‐filled teeth and clinical findings, the diagnosed conditions should be adequately treated and the results evaluated, whereby the cause of pain can be determined in retrospect, *ex juvantibus*.

## 5 Conclusion

In a cohort of adults attending regular dental care, pain from root‐filled teeth can arise from various conditions. Apical periodontitis (AP) appears to be a common source. TMD and marginal periodontitis (MP) may also contribute to or be the primary pain. Other underlying conditions may also be responsible, and investigating this would require additional and distinct diagnostic methods.

## Conflicts of Interest

The authors declare no conflicts of interest.

## Supporting information


**Supporting Information S1:** joor70089‐sup‐0001‐Supplement1.docx.

**Supporting Information S2:** joor70089‐sup‐0002‐Supplement2.docx.

## Data Availability

The data that support the findings of this study are available on request from the corresponding author. The data are not publicly available due to privacy or ethical restrictions.

## Appendix

EndoReCo collaborators: Lars Bjørndal, Victoria S. Dawson, Helena Fransson, Fredrik Frisk, Peter Jonasson, Thomas Kvist, Merete Markvart, Maria Pigg, Dan Sebring, Emma Wigsten.
