# Supplementary material for: Root‐Filled Teeth With and Without Pain in a Cohort of Individuals Scheduled for Regular Dental Check‐Ups. A Matched Case–Control Study
Source: J Oral Rehabil. 2025 Oct 28;53(2):368–78. doi: 10.1111/joor.70089 (PMC12813518; doi:10.1111/joor.70089)
Supplement: Supplementary file 2 — Supporting Information S2: joor70089‐sup‐0002‐Supplement2.docx. [file JOOR-53-368-s002.docx]

|  | | | Clinical signs that indicate AP | | | | Radiological signs that directly indicate AP | Radiological signs that indirectly indicate AP | | | | | Clinical signs of MP | Radio-logical signs indicating MP | Questionnaire data indicating TMD | Plausible pain origin for case |
| --- | --- | --- | --- | --- | --- | --- | --- | --- | --- | --- | --- | --- | --- | --- | --- | --- |
| Gender | Age | Tooth | Tenderness to percussion | Tenderness to apical palpation | Sinus tract | Swelling | Apical radiolucency | Incomplete obturation | Length of root filling >2mm short of apex, or excess | Unfilled canals | Inade-quate coronal seal | Secondary caries | Greatest probing depth ≥ 6mm | Marginal breakdown | 3Q/TMD  Positive Q1 or Q2 |  |
| Male | 29/38 | 11/12 | -/- | +/- | -/- | -/- | +/- | +/- | -/- | -/- | -/- | -/- | -/- | -/- | -/- | AP-1 |
| Male | 59/57 | 23/11 | +/- | -/- | -/- | -/- | -/- | -/+ | +/+ | -/- | -/- | -/- | -/- | +/+ | +/- | AP-3/TMD |
| Male | 71/71 | 22/13 | -/- | -/- | -/- | -/- | +/- | -/+ | -/- | -/- | -/- | +/- | -/- | +/+ | -/- | AP-2 |
| Male | 37/38 | 25/15 | -/- | -/- | -/- | -/- | -/- | -/+ | -/- | -/- | -/+ | -/- | -/- | +/- | -/- | 0 |
| Male | 41/43 | 25/14 | -/- | -/- | -/- | +/- | -/- | +/+ | +/+ | -/- | +/+ | -/- | -/- | +/- | -/+ | AP-3 |
| Male | 43/49 | 16/26 | +/- | -/- | -/- | -/- | -/- | -/- | +/+ | -/+ | -/- | -/- | -/- | +/+ | -/- | AP-3 |
| Male | 48/44 | 27/16 | -/- | -/- | -/- | -/- | +/+ | +/+ | -/+ | +/+ | -/- | -/- | -/- | +/- | -/- | AP-2 |
| Male | 42/49 | 36/46 | -/- | -/- | -/- | -/- | -/- | +/+ | +/+ | -/- | -/- | -/- | -/- | -/+ | +/- | AP-4/TMD |
| Male | 48/46 | 36/37 | -/- | -/- | -/- | -/- | +/+ | +/+ | -/+ | -/- | -/- | -/- | -/- | +/+ | -/- | AP-2 |
| Male | 58/52 | 26/27 | -/- | -/- | -/- | -/- | -/- | -/+ | -/+ | -/- | -/- | -/- | -/- | +/+ | -/- | 0 |
| Male | 58/56 | 27/16 | -/- | -/- | -/- | -/- | -/- | -/- | -/+ | -/- | -/- | -/- | +/- | +/- | -/- | MP |
| Male | 57/56 | 47/36 | -/- | -/- | -/- | -/- | -/- | -/+ | +/- | -/- | -/- | -/- | -/- | +/+ | -/- | AP-4 |
| Male | 52/59 | 46/36 | +/- | -/- | -/- | -/- | +/- | +/+ | -/- | -/- | -/- | -/- | -/+ | -/+ | -/- | AP-1 |
| Male | 60/53 | 46/46 | +/- | -/- | -/- | -/- | +/+ | -/+ | -/- | -/- | -/- | -/- | */- | +/+ | */- | AP-1 |
| Male | 68/70 | 16/16 | -/- | -/- | -/- | -/- | -/- | */+ | -/+ | -/- | +/- | -/- | -/- | +/- | -/- | AP-4 |
| Male | 64/70 | 17/26 | -/- | -/- | -/- | -/- | +/- | -/- | -/+ | -/+ | -/- | -/- | +/- | +/+ | -/- | AP-2/MP |
| Male | 62/64 | 37/36 | +/- | +/- | -/- | -/- | -/- | -/- | -/+ | -/- | -/- | +/- | +/- | +/+ | -/- | AP-2/MP |
| Male | 63/65 | 36/46 | +/- | -/- | -/- | -/- | +/- | -/+ | -/+ | -/+ | +/+ | -/- | -/- | -/+ | -/- | AP-1 |
| Male | 72/75 | 26/26 | -/- | -/- | -/- | -/- | -/- | +/- | +/- | -/- | -/- | -/- | -/- | -/+ | +/- | AP-4/TMD |
| Male | 73/79 | 46/36 | +/- | -/- | -/- | -/- | +/- | -/- | -/- | -/- | -/- | -/- | +/- | -/+ | -/- | AP-1 |
| Female | 51/55 | 11/11 | +/- | -/- | -/- | -/- | -/- | -/- | +/- | -/- | -/- | -/- | -/- | -/+ | -/- | AP-4 |
| Female | 52/57 | 21/22 | -/- | -/- | -/- | -/- | -/- | -/- | -/+ | -/- | -/- | -/- | -/- | +/+ | -/+ | 0 |
| Female | 59/55 | 11/12 | -/- | -/- | -/- | -/- | -/- | -/- | +/- | -/- | -/- | -/- | -/- | +/+ | -/- | AP-4 |
| Female | 62/66 | 22/22 | -/- | -/- | -/- | -/- | -/- | -/+ | -/- | -/- | -/- | -/- | -/- | +/+ | -/- | 0 |
| Female | 69/66 | 12/12 | +/- | -/- | -/- | -/- | +/- | -/+ | -/+ | -/- | -/- | -/- | -/- | +/+ | -/- | AP-1 |
| Female | 74/72 | 23/23 | +/- | +/- | +/- | +/- | -/- | -/+ | +/- | -/- | +/- | -/- | +/- | +/- | +/+ | AP-1/MP  /TMD |
| Female | 74/75 | 21/21 | -/- | -/- | +/- | -/- | +/- | +/+ | -/- | -/- | -/- | -/- | -/- | -/+ | -/- | AP-1 |
| Female | 71/77 | 43/32 | -/- | -/- | -/- | -/- | -/+ | -/- | -/+ | -/- | -/- | -/- | -/- | -/+ | -/- | 0 |
| Female | 82/94 | 22/23 | -/- | -/- | -/- | -/- | +/- | +/- | -/- | -/- | -/- | -/- | -/- | -/+ | -/- | AP-2 |
| Female | 36/39 | 15/15 | +/- | -/- | -/- | -/- | -/- | +/- | -/- | -/- | -/+ | -/- | -/- | -/+ | -/- | AP-3 |
| Female | 44/50 | 15/24 | -/- | -/- | -/- | -/- | -/- | -/- | +/- | -/- | -/- | +/- | -/- | +/+ | -/- | AP-4 |
| Female | 44/45 | 14/15 | -/- | -/- | -/- | -/- | +/- | +/+ | +/- | -/- | -/- | -/- | -/- | +/- | -/+ | AP-2 |
| Female | 52/60 | 34/45 | -/- | -/- | -/- | -/- | -/+ | -/+ | -/+ | -/- | -/+ | +/+ | -/- | +/+ | +/+ | AP-4/ TMD |
| Female | 64/69 | 14/15 | -/- | -/- | -/- | -/- | +/- | -/- | -/- | -/- | +/- | -/- | -/- | +/+ | -/- | AP-2 |
| Female | 67/70 | 35/34 | -/- | -/- | -/- | -/- | -/+ | -/- | -/- | -/- | -/+ | -/- | -/- | +/+ | -/- | 0 |
| Female | 70/65 | 35/35 | +/- | -/- | -/- | -/- | -/- | -/- | +/- | -/- | -/- | -/- | +/- | +/+ | -/- | AP-3/MP |
| Female | 38/35 | 26/16 | +/- | -/- | -/- | -/- | +/- | +/- | -/- | -/- | -/- | -/- | -/- | -/- | +/- | AP-1/TMD |
| Female | 21/20 | 26/16 | +/- | -/- | -/- | -/- | -/- | +/- | +/+ | -/- | -/- | -/- | -/- | -/+ | +/- | AP-3/TMD |
| Female | 37/31 | 36/36 | +/- | -/- | -/- | +/- | -/- | -/+ | -/+ | -/- | -/- | -/- | +/- | +/- | -/+ | AP-2/MP |
| Female | 47/48 | 36/37 | -/- | -/- | -/- | -/- | -/- | +/- | +/- | -/- | -/- | -/- | -/- | -/- | -/- | AP-4 |
| Female | 45/45 | 37/36 | +/- | +/- | -/- | +/- | -/- | +/- | -/+ | -/- | -/- | -/- | +/- | +/- | +/- | AP-2/MP/ TMD |
| Female | 46/45 | 46/37 | -/- | -/- | -/- | -/- | +/- | -/- | -/+ | -/- | -/- | -/- | -/- | +/- | +/- | AP-2/TMD |
| Female | 47/42 | 46/36 | -/- | +/- | -/- | +/- | -/* | +/* | +/+ | -/- | -/- | -/- | -/- | -/+ | +/- | AP-1, TMD |
| Female | 47/49 | 47/46 | -/- | -/- | -/- | +/- | -/- | -/+ | +/+ | -/- | -/- | -/- | -/- | +/+ | +/- | AP-3, TMD |
| Female | 57/52 | 47/36 | +/- | -/- | -/- | -/- | +/+ | +/- | +/- | -/- | -/- | -/- | -/- | +/- | -/- | AP-1 |
| Female | 53/53 | 46/46 | +/- | -/- | -/- | -/- | +/- | +/- | +/- | -/- | -/- | -/- | -/- | -/- | -/- | AP-1 |
| Female | 59/56 | 46/46 | +/- | -/- | -/- | -/- | -/- | -/+ | -/- | -/- | -/- | +/- | -/- | +/+ | -/- | AP-3 |
| Female | 69/67 | 16/26 | -/- | -/- | -/- | -/- | -/- | -/+ | -/+ | -/- | +/- | -/+ | -/- | +/- | +/- | AP-4/TMD |
| Female | 62/65 | 26/26 | -/- | +/- | +/- | +/- | +/- | -/+ | -/- | -/- | +/- | -/- | -/- | +/+ | +/- | AP-1, TMD |
| Female | 66/65 | 26/26 | +/- | -/- | -/- | -/- | -/- | -/+ | -/- | -/- | -/- | -/- | -/- | +/- | -/- | AP-3 |
| Female | 67/63 | 26/17 | +/- | +/- | -/- | -/- | +/- | +/- | +/- | -/- | -/- | -/- | -/- | +/+ | +/- | AP-1, TMD |
| Female | 63/61 | 46/36 | -/- | -/- | -/- | -/- | -/- | +/- | -/- | -/- | +/- | +/- | +/- | -/+ | -/- | AP-4 |
| Female | 69/61 | 46/46 | -/- | -/- | -/- | -/- | -/- | -/+ | +/+ | -/- | -/- | -/- | -/- | +/+ | +/- | AP-4/TMD |
| Female | 70/67 | 46/46 | -/- | -/- | -/- | -/- | -/- | -/+ | -/+ | -/- | +/+ | -/+ | -/- | -/+ | -/- | AP-4 |
| Female | 82/86 | 16/16 | -/- | -/- | -/- | -/- | -/+ | +/+ | -/+ | -/+ | -/- | -/- | +/- | +/+ | -/- | AP-4/MP |

Supplement 2. The clinical, radiographic, and anamnestic characteristics of the coupled cases and controls. Each cell shows the case (left) and the control (right). Asterisk denotes missing data. Abbreviations: AP = Apical Periodontitis; MP = Marginal Periodontitis; TMD = Temporomandibular Disorder; AP-1 = Definite AP; AP-2 = Probable AP; AP-3 = Possible AP; AP-4 = Conceivable AP. Beige cells represent clinical signs that indirectly indicate AP. Orange represents clinical and radiographic signs that directly indicate AP. Yellow represents radiological signs that indirectly indicate AP. Signs of MP are green, and signs of TMD are blue.
